# The Roles of Electrostatic Interactions in Capsid Assembly Mechanisms of Giant Viruses

**DOI:** 10.3390/ijms20081876

**Published:** 2019-04-16

**Authors:** Yuejiao Xian, Chitra B. Karki, Sebastian Miki Silva, Lin Li, Chuan Xiao

**Affiliations:** 1 Department of Chemistry, University of Texas, 500 West University Ave, El Paso, TX 79902, USA; yxian@utep.edu; 2 Department of Physics, University of Texas, 500 West University Ave, El Paso, TX 79902, USA; cbkarki@miners.utep.edu (C.B.K.); smikisilva@miners.utep.edu (S.M.S.)

**Keywords:** electrostatic interaction, giant virus, PBCV-1, assembly, DelPhi, binding energy, binding funnel

## Abstract

In the last three decades, many giant DNA viruses have been discovered. Giant viruses present a unique and essential research frontier for studies of self-assembly and regulation of supramolecular assemblies. The question on how these giant DNA viruses assemble thousands of proteins so accurately to form their protein shells, the capsids, remains largely unanswered. Revealing the mechanisms of giant virus assembly will help to discover the mysteries of many self-assembly biology problems. *Paramecium bursaria* Chlorella virus-1 (PBCV-1) is one of the most intensively studied giant viruses. Here, we implemented a multi-scale approach to investigate the interactions among PBCV-1 capsid building units called capsomers. Three binding modes with different strengths are found between capsomers around the relatively flat area of the virion surface at the icosahedral 2-fold axis. Furthermore, a capsomer structure manipulation package is developed to simulate the capsid assembly process. Using these tools, binding forces among capsomers were investigated and binding funnels were observed that were consistent with the final assembled capsid. In addition, total binding free energies of each binding mode were calculated. The results helped to explain previous experimental observations. Results and tools generated in this work established an initial computational approach to answer current unresolved questions regarding giant virus assembly mechanisms. Results will pave the way for studying more complicated process in other biomolecular structures.

## 1. Introduction

**Figure 1:**
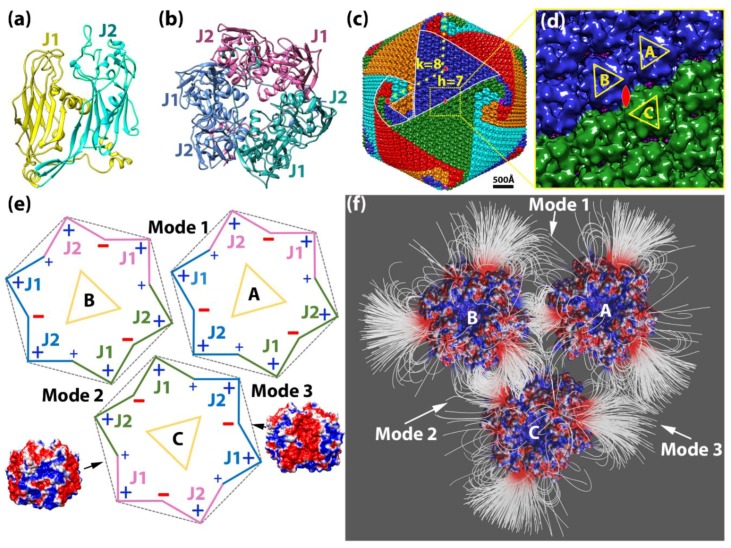
*Paramecium bursaria* Chlorella virus-1 (PBCV-1) capsomer structures and arrangements. (**a**) A ribbon diagram of the PBCV-1 major capsid protein (MCP) Vp54 (PDB ID 5TIP). The two jelly-rolls are colored in yellow (J1) and cyan (J2), respectively; (**b**) a ribbon diagram of the PBCV-1 capsomer displaying a pseudo-hexagonal shape. Individual double jelly-roll MCPs are color-coded with pink, jade green, and azure; (**c**) Isosurface rendering of PBCV-1 cryo-EM map (EMD 5378). Capsomers are colored based on their orientation in red, blue, green, cyan, and orange. The boundaries of one trisymmetron and one pentasymmetron are outlined in white. One asymmetric unit within one pentasymmetron is outlined in dashed white lines. The yellow dots present the steps for calculating the triangulation number (T number) with the h and k numbers labeled in yellow. One set of icosahedral symmetry symbols are labeled in red; (**d**) The magnified icosahedral 2-fold surface areas (outlined in yellow dashed line in (**c**)) and three selected capsomers (A, B, and C). To show their orientations, these three capsomers are labeled by yellow triangles where the vertices of the triangle point to the three higher jelly-roll surface loops; (**e**) A schematic diagram of showing the three selected capsomers in (**d**) for highlighting their orientations, charge distributions, and binding modes. The outlines of the capsomer are colored as the same color in (**b**) with J1 and J2 labeled. Three binding modes are labeled by 1, 2, and 3, close to the corresponding interfaces. Two side views of PBCV-1 capsomer electrostatic surfaces are presented on the sides of capsomer C pointing to their corresponding surface. The electrostatic surfaces are rendered by Chimera with a color scale from −1.0 to 1.0 kT/Å. Negatively and positively charged areas are colored in red and blue, respectively. (**f**) The electrostatic potential field lines between three selected PBCV-1 capsomers rendered by Visual Molecular Dynamics (VMD). Negatively and positively charged capsomer surface areas are colored with a scale from −1.0 to 1.0 kT/Å.

Since the early 1990s, many giant DNA viruses that infect eukaryotic cells have been discovered and studied [1].Examples include *Paramecium bursaria* Chlorella virus 1 (PBCV-1) [2,3], *Chilo* iridescent virus [4], *Phaeocystis pouchetii* virus [5], *Mimivirus* [6], *Megavirus* [7], *Cafeteria roenbergensis* virus [8], *Faustovirus* [9], *Sambavirus* [10], as well as non-icosahedral-shaped giant viruses [11]. Cellular genes found in all three kingdoms of life are present in giant virus genomes, reigniting the debate about the tree of life [12,13] as well as the definition of a virus [14]. The size and complexity of giant viruses have challenged current structural techniques [1] and present a unique research frontier for the study of self-assembly and regulation of supramolecular assemblies on an extremely large scale. Despite their size and complexity, the protein shell (capsid) of icosahedral giant DNA viruses still assembles symmetrically from simple and similar building blocks called capsomers. Each of the donut-shaped capsomers consists of six “jelly-roll” folds [15]. The “jelly-roll” fold is a wedge-shaped β-barrel structure that is used by all kinds of DNA or RNA viruses infecting species across all three kingdoms [16], varying in size from small to giant DNA viruses [1]. In giant icosahedral DNA viruses, two jelly-roll folds are linked together in their major capsid protein (MCP), and the capsomer is a trimer of the MCP (Figure 1a,b). These trimeric capsomers then pack closely to cover the icosahedral surface of the virus (Figure 1c). However, how giant icosahedral DNA viruses assemble their gigantic capsid from the capsomers so accurately is still largely unknown.

In 1969, Wrigley described large triangular and pentagonal arrays of capsomers resulting from broken *Sericesthis* iridescent virus (SIV) [17]. He defined these as trisymmetrons and pentasymmetrons and derived mathematical formulas for possible symmetron sizes. Later, pentasymmetrons and trisymmetrons could be clearly identified on the capsid surface by the discontinuous lines between them. This discontinuity occurs because three of the six jelly-rolls within one capsomer are positioned a little higher towards the exterior of the virus with longer surface loops (Figure 1a) in many giant viruses, conveying a truly trimeric appearance of the capsomer (Figure 1c,d). Within trisymmetrons, the capsomers are packed in the same orientation, but between neighboring trisymmetrons the orientation of capsomers differs by 60° (Figure 1d), thus generating visible discontinuous lines on the virus surface [18]. Based on all these observations, it was assumed that the giant virus capsomers first assembled into these symmetrons, which then joined together to form the capsid. However, electron tomography studies of thin sectioned cells infected by giant viruses have shown that the assembly starts from one 5-fold vertex and seems to be continuous [19]. No large patches of capsomers have been observed inside host cells. A recent close analysis of the capsomer orientations around the icosahedral 5-fold axis among several giant icosahedral giant DNA viruses revealed a spiral pattern within the traditionally defined pentasymmetron. A new spiral assembly hypothesis has been raised that is consistent with the continuous process observed inside the cells [8]. Recently, a near-atomic structure of PBCV-1 was published [20]. Thirteen different minor capsid proteins (mCPs) were identified, and an intensive network of interactions among mCPs and MCPs was observed, indicating mCPs might also play important roles in assembly. 

Computational simulations are widely used in viral capsid assembly research. Some of the simulations helped to resolve capsid structures [21], others aimed at revealing the mechanisms of viral capsid assembly [22,23,24]. Even though the large size and the complexity of the viral capsids make it extremely challenging, many computational works have been done to understand the mechanisms of virus capsid assembly [25,26]. A significant portion of these simulation works were based on coarse-grained models, which selectively captured the main information of residues instead of focusing on some atomic details in simulations. Very few atomic-level simulations were performed on a whole capsid [27]. Most of them focused on particular regions of the capsid assembly, such as the scaffold protein-mediated capsomer–capsomer interactions [28]. 

Electrostatic interaction has been recognized as an important factor for protein–protein recognition and assembly [29,30,31,32]. In previous works, we have successfully calculated and illustrated the electrostatic potential distribution and electric field lines around a whole viral capsid [33]. In this research, we investigated the possible interactions between capsomers by using PBCV-1 as the model system because of its available atomic MCP structure [34] as well as its high-resolution, intact virus density map [2]. To study the binding mechanisms of capsomers, we used simulation methodology to calculate the interactions between each two capsomers among three chosen ones around the icosahedral 2-fold area of PBCV-1 capsid. 

Three binding modes were found between capsomers with different strengths. To mimic the assembly process, we manipulated capsomer pairs in four different operations. Binding force calculation showed that electrostatic interaction was critical to guide the assembly of the capsomers. Calculated by the MM/PBSA (molecular mechanics with Poisson–Boltzmann and surface area solvation) method, the total binding energies of these three binding modes were all negative, indicating their contribution to stabilize the capsid. Furthermore, formulas were derived to calculate total numbers of different binding modes within a single capsid. One of the binding modes provided the dominating stabilization interactions, which were consistent with experimental observations.

In this study we focused on the dominant binding modes, which are located in intra- and inter-trisymmetrons regions. However, the interactions among capsomers within the pentasymmetrons are also important because they are essential for initializing the assembly of the viral capsid. Structural manipulation tools with four operations have been developed to explore the electrostatic binding forces between two capsomers in each binding mode, which pave the way for future investigation of capsomer interactions among pentasymmetrons of giant viruses. The MM/PBSA approach is based on 20 ns molecular dynamics (MD) simulations on three capsomer complexes and is the first step to investigate capsomer–capsomer interactions. The spiral pattern of capsomer orientations and the protruding shape of the 5-fold vertices of the pentasymmetron require more complicated calculations and more detailed examinations, which will be our future work.

In summary, our results demonstrated that the electrostatic interactions played crucial roles in driving the assembly as well as stabilizing the final capsid after the assembly process of PBCV-1. It is noteworthy that our work establishes the initial computational studies for the interactions among certain capsomers on relatively flat areas of the capsid. Further computations are needed before expanding to other capsomers where curvature needs to be considered. Nevertheless, these results shed the first light on the mechanisms of giant virus assembly and are a solid stepping stone for addressing more complicated assembly interactions such as those between mCPs and MCPs [20]. Furthermore, various tools developed in this work can be widely utilized in studying protein–protein interactions beyond virus capsid assembly such as other self-assembly supramolecular complexes within cells.

## 2. Results and Discussion

### 2.1. Electrostatic Potential for the Capsomers

#### 2.1.1. Single Capsomer

The charge distribution on a single capsomer showed an interesting pattern. Calculations in Delphi demonstrated that the net charge of the capsomer was −3. The charge distribution was visualized in Chimera (Movie S1). Note that negatively charged residues were predominant in between the two jelly-rolls within each MCP monomer; positively charged residues were mostly located at the interface between the neighboring MCP monomers, and the rest of the positively charged residues were located on the outside edge of each jelly-roll (Figure 1e; Movie S1). The overall charge distribution is shown in a schematic diagram (Figure 1e). Negatively charged residues were dominant in every other groove of the pseudo-hexagon, whereas positively charged residues were most dominant on the vertices. This unique feature of charge distribution of the PBCV-1 capsomer indicated that charge interactions on its side may provide strong and elegant forces to cause the biological assembly of PBCV-1 capsomers.

#### 2.1.2. Interaction between Capsomers

Within the three capsomers, an attractive electrostatic interaction was observed in binding mode 1 between capsomers A and B as well as in binding mode 2 between capsomers B and C. In binding mode 3, capsomers A and C were shown to have repulsive electrostatic interactions, as expected, since their interfaces were dominated with positively charged residues (Figure 1e). Furthermore, the electrostatic interaction in binding mode 2 between capsomers B and C was shown to be stronger with denser field lines at the interface than binding mode 1 between capsomers A and B (Figure 1f). Almost no field lines were found at the interface of mode 3, which was consistent to repulsive interaction (Figure 1f). Therefore, we referred these three binding modes as intermediate electrostatic-attractive binding (mode 1), strong electrostatic-attractive binding (mode 2), and electrostatic-repulsive binding (mode 3).

#### 2.1.3. Salt Bridges Contribute to the Electrostatic Interaction between Capsomers

At the interfaces of binding modes 1, 2, and 3, a total of four, two, and two salt bridges were found during MD simulations, respectively (Table S1). For each binding mode, the total salt bridge number within each frame was also analyzed in order to determine the salt bridge population (Figure S2a,b,c). During MD simulations, binding mode 3 had the lowest average number of salt bridges. This result was consistent with the electrostatic potential calculations. However, binding mode 1 seemed to maintain a higher salt bridge population than binding mode 2, even though the electric field lines showed that binding mode 2 had stronger binding interactions. It is noteworthy that we only counted those salt bridges with a distance of 4 Å. Other charged interactions beyond this distance might still significantly contribute to the electrostatic interactions in binding mode 2, which can be verified by quantitative calculations in later sections on binding force and binding energy calculations. On the other hand, this observation also showed that at close contact distance, mode 1 had more stable complimentary charged residue pairs compared to mode 2. 

### 2.2. Electrostatic Binding Force between Capsomers 

**Figure 2:**
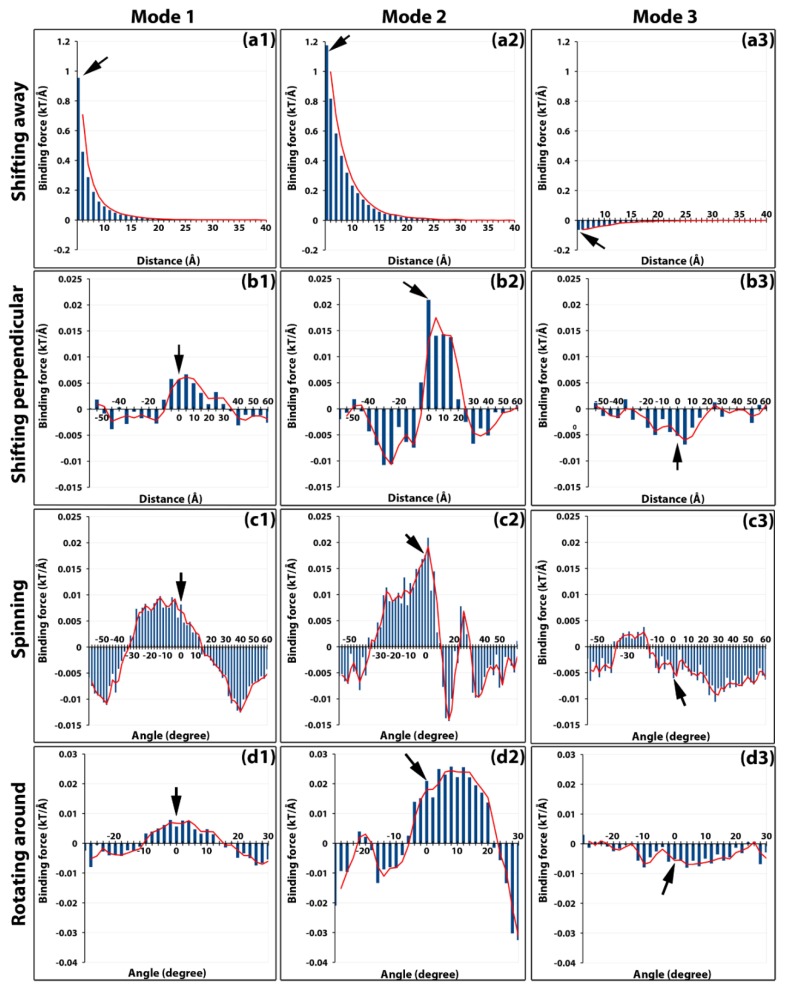
Binding forces of modes 1, 2, and 3 scanned by all four operations. The magnitudes of binding forces are presented as vertical histogram bars for each binding mode (panels in each column). Binding forces of each mode are scanned by four different operations (panels in each row). For example, the panels in the first column are binding forces of mode 1 scanned by (**a1**) shifting away 5 to 40 Å, (**b1**) shifting perpendicular 5 to 60 Å up and down, (**c1**) spinning −60° to 60°, and (**d1**) rotating around −30° to 30°. Similarly, the panels in the second column (**a2**, **b2**, **c2**, **d2**) and that in the third column (**a3**, **b3**, **c3**, **d3**) are binding forces of mode2 and mode3, respectively. Positive forces indicate the attractive force while the negative forces are the repulsive forces. Black arrows on each panel point to the native position.

To compare the strengths of the binding forces in all three binding modes, the calculated total forces versus the distance from 5 to 40 Å for each binding mode were plotted (Figure 2a). This clearly demonstrated that the binding forces in binding modes 1 and 2 were attractive and in binding mode 3 were repulsive, consistent with the electric field line analyses above (Figure 1f). Furthermore, the attractive force in binding mode 2 was stronger, approximately 1.2 to 2.0 times at various distances, than that of binding mode 1. The maximum magnitude of repulsive mode 3 was 1/20 to 1/10 of that of mode 1, indicating its repelling contribution was minor. In addition to the magnitudes, the directions of the binding forces at different distances were plotted in Figure S3. For binding modes 1 and 2, the forces were attractive up to 35 Å, beyond which the directions of the forces became random (Figure S3) while the magnitudes became insignificant (Figure 2a). This indicated the range of effective forces of modes 1 and 2 was around 35 Å, which meant that during the viral capsid assembling, capsomers needed to be brought together into this range when the electrostatic binding forces became effective.

For perpendicular shifting operations from −60 to 60 Å for all three binding modes, global maximum attractive forces for modes 1 and 2 and repulsive force for mode 3 were all located near the native position (Figure 2b). The distribution of the forces showed attractive funnels between −10 to +35 Å and between −5 to 25 Å for mode 1 and 2, respectively (Figure 2b1,b2). Overall for mode 3, the repulsive forces formed a funnel ranging from −35 to +25 Å and was centered near the native position (Figure 2b3).

For spinning operations of binding mode 1, one of the strongest forces exerted toward the fixed capsomer was observed in the native orientation. The range of attractive forces funnel was from −30° to 10° (Figure 2c1). For binding mode 2, the native orientation had the strongest binding force with the attractive funnel ranging from −40° to 10° (Figure 2c2). For binding mode 3, consistent with all our previous observations, most of the binding forces were repulsive with the strongest one at around 30° (Figure 2c3).

The binding forces of rotating capsomer B around A in binding mode 1 showed the binding funnel width was 25°, from −10° to 15°. The center of the binding funnel was located near the native position (Figure 2d1). For binding mode 2, one of the strongest attractive binding forces was found at the native position. The range of the attractive funnel was from −5° to 25° (Figure 2d2). For binding mode 3, again consistent with all our previous observations, the binding forces remained mostly repulsive with two peaks located at −10° and 5°. The native position was near one of the peaks (Figure 2d3).

For each binding mode, binding funnels were investigated in various perspectives. These calculations of the effects of distance and orientation to the binding force showed that when the manipulated capsomer was placed at a relatively close distance, within 35 Å to the fixed one, the electrostatic forces not only attracted them together but also adjusted their orientations to achieve optimal binding for the assembly.

Based on the capsomer orientations (Figure 1d,e), within the trisymmetron, individual capsomers were assembled using three mode 1 interactions, which were all attractive. In contrast, at the boundaries of the trisymmetrons, individual capsomers had one intermediate attractive (mode 1), one strong attractive, and one repulsive interaction. In summary, in either situation, each capsomer had similar overall binding affinity to its surrounding capsomers, which was further confirmed by the energy calculations discussed below.

### 2.3. Binding Energy Calculation Results from Molecular Mechanics with Poisson–Boltzmann and Surface Area Solvation (MM/PBSA) Analyses

Binding free energy was calculated to be −60.85 kcal/mol for binding mode 1, −98.43 kcal/mol for binding mode 2, and −23.81 kcal/mol for binding mode 3 (Table 1). Overall, all three binding modes were shown to have attractive binding and would contribute to stabilize the viral capsid during and after its assembly. The Coulombic components were consistent with the electrostatic forces discussed above, that binding mode 2 was strongly attractive (−167.72 kcal/mol), binding mode 1 was weakly attractive (−64.89 kcal/mol), and binding mode 3 was repulsive (23.69 kcal/mol). In the electrostatic-repulsive binding mode 3, even though the charged residues in the interface were repelling each other, the total binding free energy was still negative mainly because non-polar solvation parts and van der Waals energy were present. Binding mode 2 was shown to have the strongest binding. However, at the boundary of trisymmetrons, the combined energy of binding mode 2 with the weak binding mode 3 was similar to the summation of two binding energies from mode 1. Therefore, assembly using binding mode 2 and 3 was reasonable at the boundary of trisymmetrons. After assembly, these two binding modes did contribute to the stabilization of the capsid. However, when disassembling, because mode 3 was the weakest mode with repulsive electrostatic interaction, the binding mode 3 interfaces along the boundaries of trisymmetrons should be detached first. This further indicated that the boundaries of the trisymmetrons should be the breaking lines, which provided explanation for the observations that viral capsids disassembled into trisymmetrons in previous experiments [17].

### 2.4. T-Number and Total Contribution of Each Mode in the Virion of Giant Viruses

**Figure 3:**
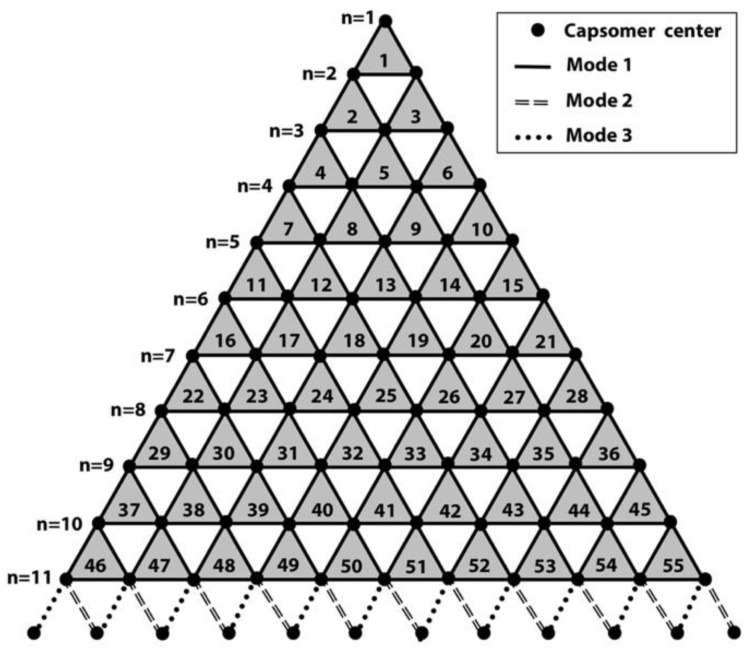
Schematic demonstration of the trisymmetron for calculating the population of all 3 binding modes. Each black dot represents one capsomer. Different types of lines represent different binding mode between two capsomers (dots). Within the trisymmetron, *n* (for PBCV-1, *n* = 11) rows of capsomers were connected by binding mode 1 (solid line). At the bottom edge, extra row of dots (capsomers) from neighboring trisymmetrons are shown connected by a dashed line and dotted lines, representing binding modes 2 and 3, respectively. The capsomer numbers along one edge are labeled by their number of *n*.

Caspar and Klug developed a mathematical formula, the triangulation number (T number), to describe the assembly geometry of icosahedral viruses [35]. The T-number is a measure of how many monomeric triangle areas (e.g., jelly-roll domains) exist in one asymmetric unit of an icosahedron. Virus surfaces are tiled by triangular areas, most of which form hexameric shapes such as the double jelly-roll capsomer on the giant virus surface (Figure 1b,c). At the 5-fold axis of the icosahedral virus, there is a pentameric capsomer that only has five triangles (jelly-rolls). By “stepping” along capsomers from one 5-fold capsomer to the nearest one (Figure 1c) in the hexagonal array, the shortest route is to take h steps along one hexagonal axis (*h*), and k steps along the other hexagonal axis (*k*). The T-number can then be calculated using the equation: *T* = *h*^2^ + *h*∙*k* + *k*^2^. For example, the T-number of PBCV-1 is 169 (*h* = 7, *k* = 8; *T* = 7^2^ + 7 × 8 + 8^2^) (Figure 1c). It is noteworthy that all giant viruses have their *h* number equal to 7 and have the same size pentasymmetrons with 31 capsomers: one pentameric capsomer located at the 5-fold axis and 30 (5 × 6) pseudohexameric capsomers [8]. Each asymmetric unit (triangular area encircled by white dotted lines in Figure 1c) inside the pentasymmetron has six capsomers in three layers radiating from the 5-fold axis, one in the first layer (closest to the 5-fold axis), two in the second layer, and three in the third layer. Therefore, the *h* number includes three steps in the pentasymmetron, three steps in the trisymmetron, and one more step to align with the pentameric capsomers (Figure 1c). The size difference among giant viruses only appears on their various sizes of trisymmetron [8]. The size of trisymmetron is linked to the k in the T-number. If the equilateral trisymmetron has *n* capsomers on its edge, because of the same three layers in a pentasymmetron asymmetric unit, *n* will be *k* plus 3 (Figure 1c and Figure 3).

Capsomers inside a trisymmetron with the same orientation interacted with each other in binding mode 1, while capsomers at the boundary of trisymmetrons interacted with each other in binding mode 2 or 3. Using PBCV-1 trisymmetron as an example (Figure 3), we can derive the formulas for the total number *N* of each mode of interactions within one virion.

(1) $$N_{mode1}=n\cdot \left(n-1\right)\cdot \frac{3}{2}$$

For binding modes 2 and 3 at each edge of trisymmetron, the number of their interaction per edge is *n*. 

(2) $$N_{mode2/3}=n$$

Because each icosahedron giant virus has 20 trisymmetron, 30 trisymmetron edges, and as aforementioned, *n* = *k* + 3, formulas of total number of each mode for one intact virion can be derived as:

(3) $$N_{mode1}=n\cdot \left(n-1\right)\cdot 3\cdot 20/2=\left(k+3\right)\cdot \left(k+2\right)\cdot 30$$

(4) $$N_{mode2/3}=n\cdot 30=\left(k+3\right)\cdot 30$$

Based on these formulas, the total number of binding in modes 1, 2, and 3 in PBCV-1 were 12,600, 540, and 540, respectively. Clearly, binding mode 1 was the dominant interaction that stabilized the final assembled virion.

## 3. Methods

### 3.1. Capsid Structure Preparation

The capsomer structure was downloaded from the Protein data Bank (PDB ID 5TIP [34]). The highest resolution cryo-EM density map of PBCV-1 was downloaded from EMDataBank (Accession code 5378 [2]). The pseudo-atomic structure of PBCV-1 capsid was generated by fitting the capsomer structure into the cryo-EM map using the fit in map function in Chimera [36]. To study the electrostatic interactions, three capsomers around the icosahedral 2-fold area were selected (Figure 1d), which represented all the possible intra- and inter-trisymmetron interactions. Capsomer A and capsomer B were from the same trisymmetron with the same orientation. Capsomer C was from the neighboring trisymmetron and was 2-fold symmetric with capsomer A. However, because of the C3 symmetry of the capsomer, capsomer C’s orientation could be described equivalently as 60°, 120°, or 180° rotated to capsomer A. Because of the special orientation of capsomer C, it could be predicted that there were three types of interactions within the selected three capsomers: binding mode 1 between capsomers A and B, binding mode 2 between capsomers B and C, and binding mode 3 between capsomers A and C (Figure 1e).

### 3.2. DelPhi Calculations of Electrostatic Potential

The electrostatic potential maps (phimap) of capsomers were generated by Delphi [37,38]. (See supplementary information for parameters and calculation details.) The calculated electrostatic potential on the surface was visualized with Chimera (Figure 1e). In order to visualize interactions between capsomers, electric field lines were shown in Visual Molecular Dynamics (VMD) [39] (Figure 1f). The color scale range was set from −1.0 to 1.0 kT/Å. In order to illustrate the field lines clearly, the distance between selected capsomers was increased by 20 Å for electrostatic potential calculations and field line demonstrations.

### 3.3. Capsomer Manipulations to Simulate Capsid Assembly

**Figure 4:**
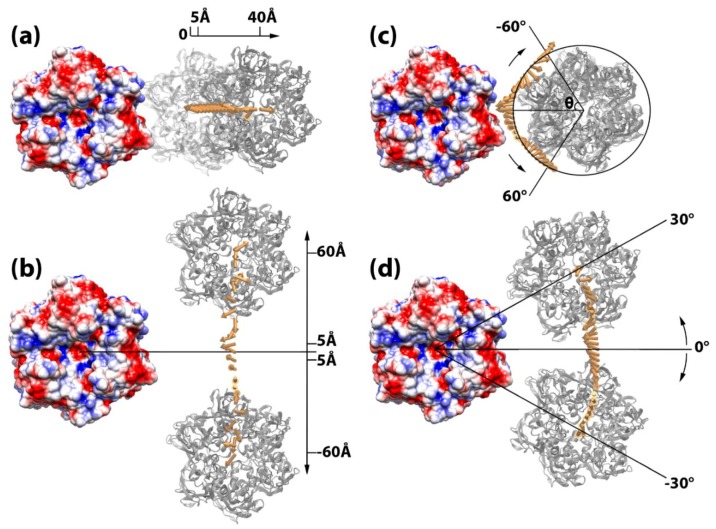
Simulating capsomer assembly operations. In each of the four operations, the capsomer on the left (shown in electrostatic colored surface) is fixed, whereas the capsomer on the right (in grey) is manipulated by (**a**) shifting away 5 to 40 Å, (**b**) shifting perpendicular 5 to 60 Å up and down, (**c**) spinning −60° to 60°, and (**d**) rotating around −30° to 30°. The binding forces are presented by yellow arrows. The tail of each arrow is located at the mass center of the manipulated capsomer when it was displaced in the corresponding locations. In order to differentiate the binding force from different spinning degree in (**c**), the force arrows are translated by 40 Å onto a circle where the spinning degrees are represented by the angle theta (θ). Additional diagrams for the direction of binding force are shown in Figure S3.

A package of structure manipulation tools has been developed and used to manipulate the capsomer pairs and mimic the assembly process. Within each pair of capsomers, one capsomer was fixed while the other one was manipulated. For the purpose of this study, which focused on capsomers with relatively flat surfaces around the icosahedral 2-fold area, four different types of geometric operations of each capsomer pair were carried out in a plane that was parallel to the surrounding flat capsid surface (Figure 4): (a) shifting away, where the manipulated capsomer was shifted away from a fixed one in the direction along the vector of the center of mass of the two capsomers (Figure 4a) from 5 to 40 Å in 1 Å intervals; (b) shifting perpendicular, where the manipulated capsomer was shifted along a vector that was perpendicular to the vector connecting the two mass centers from −60 to 60 Å, in 5 Å intervals, after shifting 20 Å (the approximate depths of the capsomer grooves) away from the fixed capsomer (Figure 4b); (c) spinning, where the manipulated capsomer was spun from −30° to 30° in 2° intervals with the respect to its own mass center, after being shifted 20 Å away to avoid atom clashes from fixed capsomers (Figure 4c); and (d) rotating around, where the manipulated capsomer was shifted 20 Å away and then rotated around the fixed capsomer in the *xy*-plane (Figure 4d) from −30° to 30° in 2° intervals. For operations of binding modes 2 and 3, capsomer C was manipulated while capsomers A or B were fixed, because capsomers A and B were within the same trisymmetron. 

### 3.4. Electrostatic Binding Forces between Capsomers

To investigate the roles of electrostatic interactions in guiding the assembly of the viral capsid, electrostatic binding forces in all three binding modes were calculated using DelphiForce [40,41]. As mentioned above, the structures in each binding mode were obtained by displacing the manipulated capsomer in various distances and orientations from the fixed capsomer utilizing the four operations (Figure 4). For example, for binding mode 1, by shifting the capsomer away from fixed capsomer from 5 to 40 Å in 1 Å intervals, 36 structures were obtained. The electrostatic force on the moving capsomer exerted from the fixed capsomer in each structure was calculated by DelPhiForce and compared so that the effects of capsomer distance could be analyzed (Figure 4a and Figure S3a). Similarly, using the other operations, the effects of both translation and rotation were investigated (Figure 4 and Figure S3). In DelPhiForce calculations, parameters and the boundary conditions were set the same as DelPhi calculations in the earlier section. Parallel operations and calculations were carried out with all three binding modes. The electrostatic binding forces calculated by DelphiForce were visualized with VMD and represented by arrows (Figure 4 and Figure S3). 

### 3.5. Molecular Dynamic (MD) Simulations for Capsomer Pairs

To predict the dynamic interactions between capsomers in all the three binding modes, MD simulations using an implicit generalized Born (GB) model were carried out using the CHARMM force field (See supplementary information for the details). [42]. Four thousand frames were saved from each of the 20 ns simulations. To analyze the roles of electrostatic interactions, the salt bridges that formed within 4.0 Å distance by the interfacial residues from each binding mode were extracted from the 4000 frames of simulation. The strongest salt bridges in each binding mode were determined by calculating their formation frequency during MD simulation (Table S1 and Figure S1). Simulations for all three binding modes are shown in Movies S2–4.

### 3.6. Binding Energy Calculations Using MM/PBSA

For the energy calculations of each binding mode, the well-developed MM/PBSA method was utilized to calculate the average binding energy from 2000 frames, which were extracted from the last 10 ns trajectory of each MD simulation (See supplementary information for more details).

## 4. Conclusions

In this work, we investigated capsomer–capsomer interactions at the icosahedral 2-fold region of PBCV-1 capsid. We demonstrated that because of the geometrical symmetry of the capsomer and charge distribution, there were three binding modes: binding mode 1, the dominant binding mode in the whole capsid, which resided inside the trisymmetron where capsomers were packed in the same orientation, and binding modes 2 and 3 were formed at the boundary between two trisymmetrons where the capsomers had different orientations. The electrostatic interactions in all the binding modes were quite different. For binding mode 1, the electrostatic interaction was attractive. However, binding modes 2 and 3, which were at the boundary of two trisymmetrons, resulted in attractive and repulsive electrostatic interactions, respectively. The salt bridge analyses demonstrated that several key residues from the binding interfaces might have played significant roles for the capsomer interactions in PBCV-1 by forming stable inter-capsomer salt bridges. Simulations showed binding mode 1 formed the highest average number of stable salt bridges (Table S1 and Figure S2). For instance, Asp324.B formed two salt bridges with a very high frequency. 

In order to verify the importance of these residues for capsomer intermolecular interactions, these residues can be mutated and further studied by simulations and experiments in the future. Combining our novel structure manipulation tool with DelPhiForce, the net forces between two capsomers in each binding mode are studied and it is shown that in binding modes 1 and 2, the electrostatic attractive binding forces are responsible for capsomer recognition. Binding funnels are found at the binding interfaces when manipulating capsomer pairs in four different ways. Such binding funnels guide and rotate two capsomers towards the final assembled positions. The results demonstrate that electrostatic forces play vital roles in viral capsid assembly by guiding the capsomers to interact with each other at the favored distance and orientation to maintain stable assembling.

Overall, when considering total binding energies, all three binding modes were shown to have attractive binding interactions, among which binding mode 2 was the strongest (Table 1). Even in electrostatic-repulsive binding mode 3 (Figure 1e,f), the total binding energy was still negative mainly because of the contributions of the non-polar part of the solvation and Van der Waals energies. Although binding mode 1 had an intermediate binding energy (unlike binding modes 2 and 3 that only existed at the boundary between symmetrons), its total numbers and more stable salt bridges (Table S1 and Figure S2) made it the dominating binding mode that stabilized the capsid during and after the assembly. These results explain the phenomenon observed by Wrigley in 1969 where large icosahedral dsDNA viruses could be decomposed into trisymmetrons [17]. Furthermore, all viruses need to disassemble during the infection process. A system dominated with intermediate binding energy and relatively weak interactions at certain areas might be energetically more favorable for virus assembly–disassembly process. Therefore, our studies illuminated the elegant mechanisms of giant viral capsid assembly and disassembly from the aspects of energy and force. 

In this study we focused on dominant binding modes, which are located in intra- and inter-trisymmetrons regions. However, the interactions among capsomers within the pentasymmetrons are also important because they are essential for initializing the assembly of the viral capsid. Structural manipulation tools with four operations have been developed to explore the electrostatic binding forces between two capsomers in each binding mode (Figure 4), which pave the way for future investigation of capsomer interaction among pentasymmetrons of giant viruses. The spiral pattern of capsomer orientations and the protruding shape of the 5-fold vertices of the pentasymmetron require more complicated calculations and more detailed examinations, which will be our future work.

## Figures and Tables

**Table 1:** Binding energy for three binding modes.

Binding Mode	Polar Solvation Energy (kcal/mol)		Non-Polar Solvation Energy (kcal/mol)		Van der Waals Energy (kcal/mol)		Coulombic Energy (kcal/mol)		Total Binding Energy (kcal/mol)	
	Mean	SD ^[a]^	Mean	SD	Mean	SD	Mean	SD	Mean	SD
1	99.41	19.46	−14.58	0.73	−80.80	6.50	−64.89	25.22	−60.85	8.05
2	208.20	28.27	−19.17	1.05	−119.74	10.25	−167.72	32.72	−98.43	9.83
3	8.42	16.23	−9.67	0.71	−46.25	5.59	23.69	16.67	−23.81	5.65

^[a]^ Standard deviation.

## Supplementary Materials

Supplementary materials can be found at https://www.mdpi.com/1422-0067/20/8/1876/s1.
