# Supplementary material for: The Roles of Electrostatic Interactions in Capsid Assembly Mechanisms of Giant Viruses
Source: Int J Mol Sci. 2019 Apr 16;20(8):1876. doi: 10.3390/ijms20081876 (PMC6514965; doi:10.3390/ijms20081876)
Supplement: Supplementary file 1 [file ijms-20-01876-s001.zip › Supplementary information.pdf]

# The roles of electrostatic interactions in capsid assembly mechanisms of giant viruses

**Yuejiao Xian[1†], Chitra B. Karki[2†], Sebastian Miki Silva[2], Lin Li[2*], and Chuan Xiao[1*]**

Correspondence to: Chuan Xiao (E-mail: cxiao@utep.edu); Lin Li (E-mail: lli5@utep.edu)

*[1]Department of Chemistry, University of Texas at El Paso. 500 West University Ave, TX,79902, USA.*

*[2]Department of Physics, University of Texas at El Paso. 500 West University Ave, TX,79902, USA.*

*[*]To whom correspondence should be addressed.*

*[†]Both authors contributed equally to this work.*

## S1. Electrostatic calculation by Dephi

Electrostatic potentials were calculated using DelPhi (The PQR file of each capsomer was generated by PDB2PQR.[1] The protonation states of titratable functional groups were assumed to be standard, corresponding to pH = 7.0. To obtain the electrostatic potential and electric field at each desired position, the electrostatic potential map (phimap) of a single capsomer was generated by Delphi[2] and shown in Movie 1. During DelPhi calculations, the resolution was set as 2 grids/Å. The dielectric constants were set as 2.0 for protein and 80 for water environment, respectively. The protein filling percentage of Delphi calculation box (perfil) was set to be 70. The probe radius for generating molecular surface was 1.4 Å. Salt concentration was set as 0.15 M. The boundary condition for the Poisson Boltzmann equation was set as dipolar boundary condition. The calculated electrostatic potential on surface was visualized with Chimera[3] (Figure. 1e). VMD[4] was used to illustrate electric field lines between capsomers (Figure. 1f). Color scale range was set to be from -1.0 to 1.0 kT/Å. In order to visualize the field lines clearly, the distance between selected capsomers was increased by 20 Å for electrostatic potential calculation and field line demonstration.

## S2. Molecular dynamic simulation

During each simulation, we set all interfacial residues to be flexible and other residues constrained using a harmonic constraint energy function with a scaling factor of 1 (Supplementary Figure. S1). An interfacial residue is defined as a residue which has at least one contact with any atoms on the other capsomer. Such constraint saves CPU time significantly. Then we performed a 20 ns MD simulation for each binding mode (2.0 fs per step, 10 million steps total). In all the simulations, the temperature of the system was set as 300 Kelvin and the pressure was set as standard using the Langevin dynamics. For each simulation, frames were saved every 2,500 step, therefore, a total of 4,000 frames were saved in each simulation trajectory.

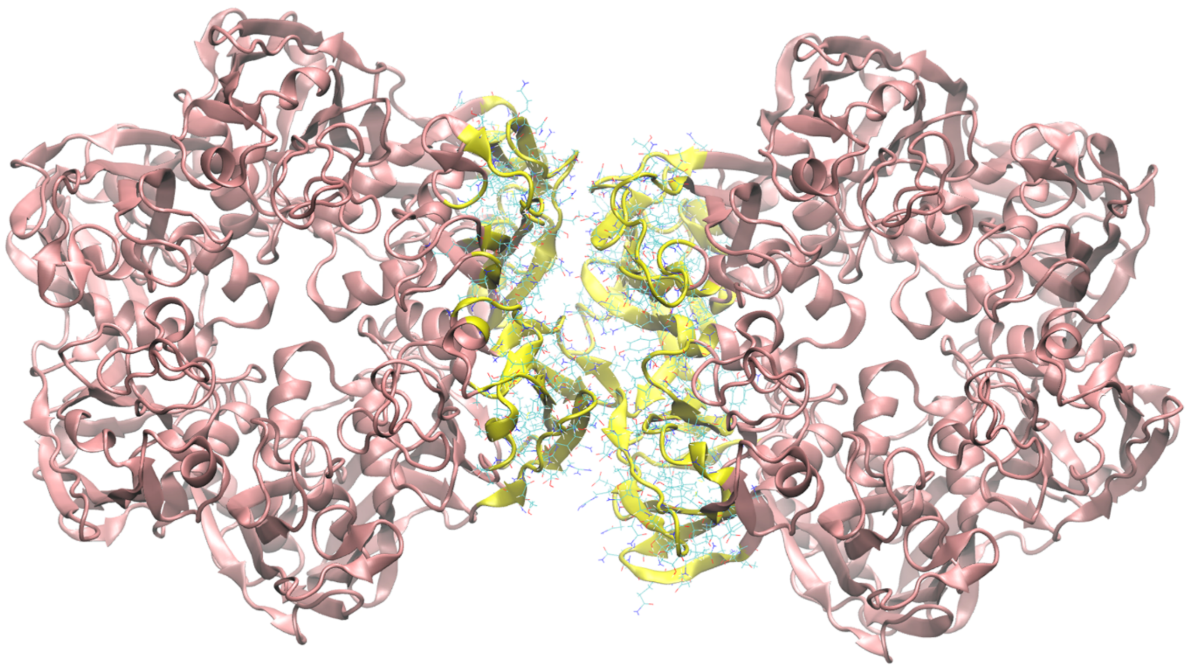

**Figure S1**. Constrained NAMD simulation. The interface residues (colored in yellow) are set free and other residues (pink) are constrained.

## S3. Energy calculation with MM/PBSA method

In this method, $\Delta G_{\text{bind}}$ is calculated as:

$$\mathrm{G}_{bind} = \mathrm{G}_{complex} - \mathrm{G}_{cap1} - \mathrm{G}_{cap2} \qquad (1)$$

Where $G_{\text{Complex}}$ is the total energy of complex of a capsomer pair; $G_{\text{cap1}}$ and $G_{\text{cap2}}$ are the total energies of the two individual capsomers. The total energy is calculated as:

$$\mathrm{G}_{total} = \mathrm{G}_{coul} + \mathrm{G}_{polar} + \mathrm{G}_{vdw} + \mathrm{G}_{\text{nonpolar}} \qquad (2)$$

Where $G_{\text{coul}}$ is the Coulombic energy, $G_{\text{polar}}$ is the polar part of the electrostatic energy, $G_{\text{vdw}}$ is the Van der Waals energy, and $G_{\text{nonpolar}}$ is the nonpolar part of the solvation energy. The Coulombic and polar electrostatic energies were calculated by DelPhi. The Van der Waals binding energy was calculated via NAMD. The nonpolar term of solvation energy was calculated via the solvent accessible surface area method:

$$\mathrm{G}_{\text{nonpolar}} = \gamma\,\mathrm{SA} + \mathrm{b} \qquad (3)$$

Where $\gamma = 0.0054 kcal \cdot mol^{-1} \cdot A^{-2}$, $b = 0.92 kcal \cdot mol^{-1} \cdot A^{-2}$, and SA denotes the solvent accessible surface area, which is calculated using Naccess2.1.1 program (http://www.bioinf.manchester.ac.uk/naccess/)

## S4. Salt bridge analysis

Table S1. Salt bridges formed with the 3 binding modes during MD simulation

| Binding modes | Salt bridges | Number | Percentage | Average[a] |
|---|---|---|---|---|
| Mode 1 | Asp324.B-Lys107.A | 1,934 | 48.35% | 1.85 |
| | Asp324.B-Arg104.A | 3,604 | 90.10% | |
| | Asp375.A-Arg252.B | 834 | 20.85% | |
| | Glu172.A-Arg62.B | 865 | 21.63% | |
| Mode 2 | Glu97.B-Arg104.C | 3,970 | 99.25% | 1.57 |
| | Glu99.C-Arg104.B | 2,226 | 55.65% | |
| Mode 3 | Asp375.C-Arg62.D | 1,670 | 41.75% | 0.59 |
| | Asp324.E-Lys317.C | 684 | 17.10% | |

[a] the average salt bridge number for each frame calculated from 4,000 frames.

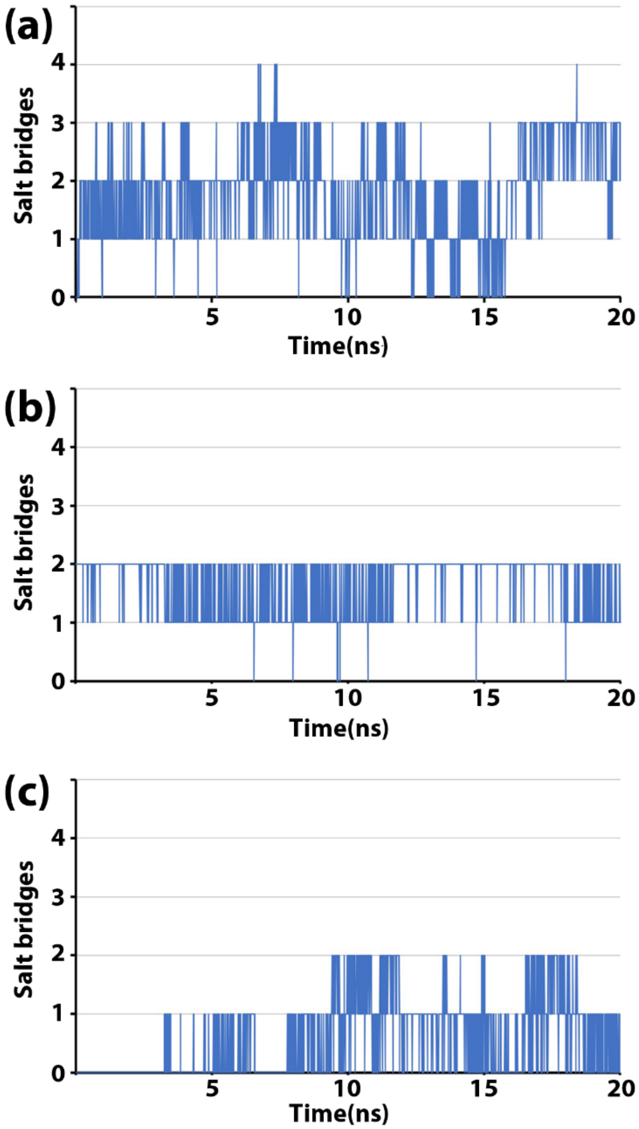


**Figure S2.** Key residues forming salt bridges at capsomer interfaces. The two capsomers are labeled in blue (left) and pink (right) to show the capsomer interface. (a), (c), and (e) show the strongest salt bridge found in modes 1, 2 and 3, respectively. (b), (d), and (f) show the total number of salt bridges counted at each frame during the 20 nm simulation for the interface of modes 1, 2 and 3, respectively.

## S5. DelphiForce Directions

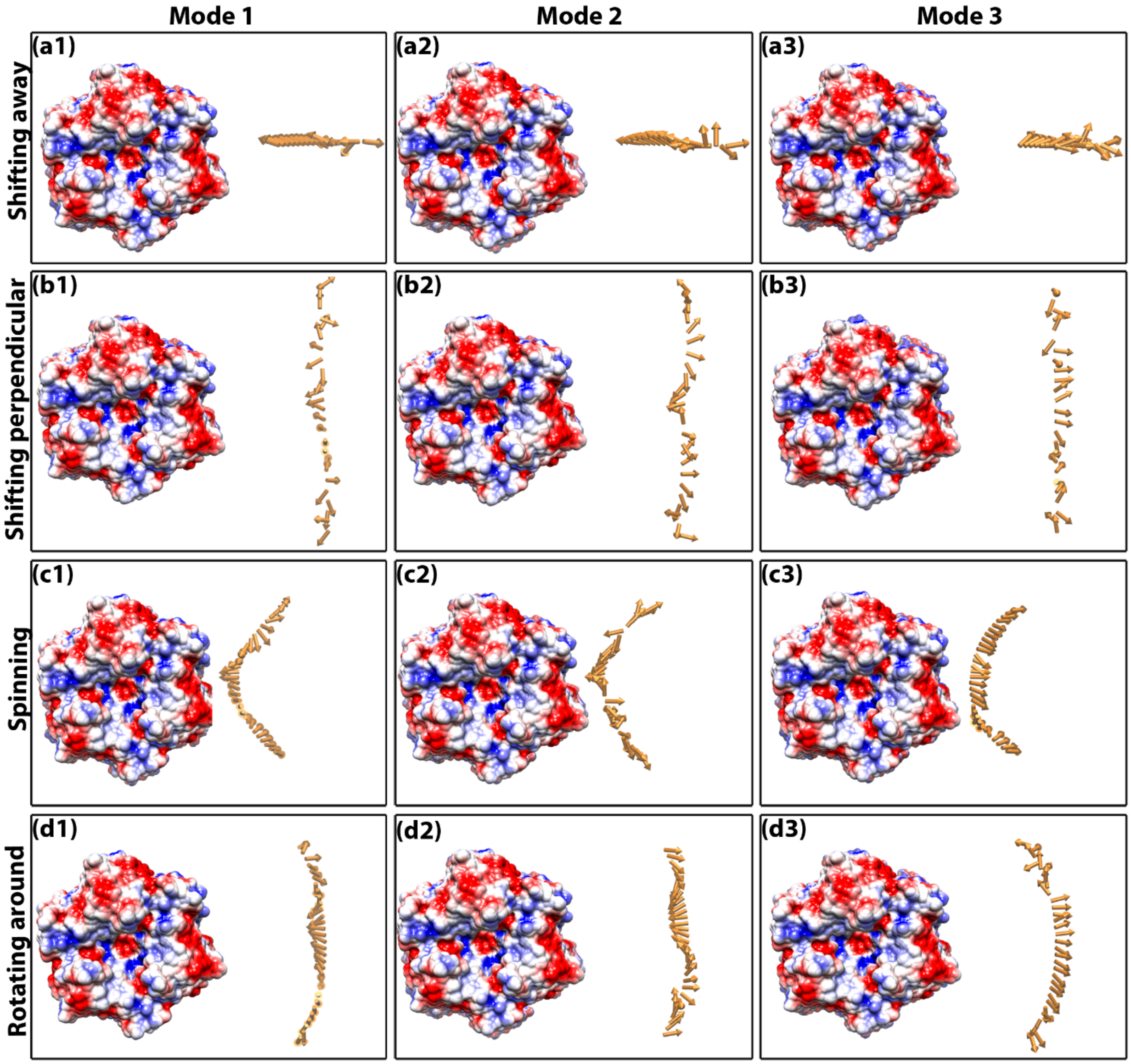


**Figure S3**. Directions of Electrostatic forces calculated with DelPhiForce. To clearly present electrostatic forces, only fixed capsomers are shown on the left. Electrostatic forces are presented as yellow arrows whose tail are located at the mass center of the manipulated capsomers (not shown) when they are manipulated by each row (a) shifting away 5 Å to 40 Å, (b) shifting perpendicular 5 Å to 60 Å up and down, (c) spinning -60° to 60°, and (d) rotating around -30° to 30° (see Figure. 2). To differentiate the binding force from different spinning degree in row (c), the force arrows are translated by 40 Å onto a circle where the spinning degrees were represented by the angles. The three columns and correspondent sub panel number represent the binding mode. Binding forces of each mode are scanned by four different operations (panels in each row). For example, panels in the first column are binding forces of mode 1 scanned by (a1) shifting away, (b1) shifting perpendicular up and down, (c1) spinning, and (d1) rotating around. To only see the force directions, all the force arrows were normalized to the same size to make the weak forces visible (especially for binding mode 3). The amplitudes of the forces are presented in Figure. 4.

## Reference of Supplementary Information


1. Dolinsky, T. J.; Nielsen, J. E.; McCammon, J. A.; Baker, N. A. Nucleic acids research 2004, 32(suppl_2), W665-W667.
2. Li, L.; Li, C.; Sarkar, S.; Zhang, J.; Witham, S.; Zhang, Z.; Wang, L.; Smith, N.; Petukh, M.; Alexov, E. BMC biophysics 2012, 5(1), 9.
3. Goddard, T. D.; Huang, C. C.; Ferrin, T. E. Journal of structural biology 2007, 157(1), 281-287.
4. Humphrey, W.; Dalke, A.; Schulten, K. Journal of molecular graphics 1996, 14(1), 33-38.
